# The reporting outcomes in medical education (ROME) model: proposition of a new framework

**DOI:** 10.1186/s12909-026-08579-z

**Published:** 2026-01-12

**Authors:** Janna-Lina Kerth, Ronny Lehmann, Jeffery W. Wells, Eleonora Leopardi, Rebecca L. Morgan, Melissa Neubacher, Juan José Yepes-Nuñez, Hans Martin Bosse

**Affiliations:** 1 Department of General Pediatrics, Pediatric Cardiology and Neonatology, Medical Faculty, University Children’s Hospital Düsseldorf, Heinrich Heine University, Moorenstrasse 5, Düsseldorf, 40225 Germany; 2 Center for Pediatrics and Adolescent Medicine, University Hospital Heidelberg, Heidelberg, Germany; 3 Department of Otolaryngology, Head and Neck Surgery, Cleveland Clinic, Cleveland, Ohio USA; 4 The Academy for Medical Education, The University of Queensland Medical School, Brisbane, Australia; 5 Department of Population and Quantitative Health Sciences, School of Medicine, Case Western Reserve University, Cleveland, Ohio USA; 6 Department of Health Research Methods, Evidence and Impact, McMaster University, Hamilton, ON Canada; 7 Department of Obstetrics and Gynecology, Medical Faculty, University Hospital Düsseldorf, Heinrich Heine University, Düsseldorf, Germany; 8 School of Medicine, Universidad de los Andes, Bogotá D.C., Colombia; 9 Fundación Santa Fe de Bogotá, Bogotá D.C., Colombia

**Keywords:** Education, Medical, Outcome Assessment (Health Care), Program Evaluation, Educational Measurement, Health Knowledge, Attitudes, Practice, Clinical Competence, Curriculum Teaching/methods

## Abstract

**Background:**

Outcomes in medical education are as complex as medical reality, ranging from individual skilled, ethical and professional development to improvement in patient care, changes in overall quality of care or changes in policy. There have been several suggestions for reporting outcome levels in medical education research, however none of which have been widely implemented,. All current models have substantial shortcomings. They lack granularity to adequately represent the complexity of medical education and care, particularly when reporting results related to patient care and health policy. Furthermore, none of the models allow for the reporting on narrative-only descriptive reports where no outcomes have yet been formally measured such as project reports.

**Methods:**

In a modified double diamond approach, we developed a new, comprehensive model taking into account the strengths and addressing the weaknesses of prior models. The preliminary model incorporated all potential outcomes in medical education without implying a hierarchy. We sent the draft model to a group of raters for testing who discussed, revised, and reviewed it. The group of raters were experienced professionals from medical education and other fields, and represented different countries (Australia, Canada, Colombia, Germany, United States).

**Results:**

The raters agreed that the proposed model was easy to understand and use and had high face validity. All raters agreed on a revised model unanimously. The final comprehensive model – ROME (Reporting Outcomes in Medical Education) model – allows for reporting both narrative and quantitative outcomes in medical education literature. We developed a corresponding Excel sheet to help with the assessment of studies as well as two worked examples to illustrate its use.

**Conclusion:**

The ROME model is easy to use and at the same time comprehensively depicts all potential outcomes potentially addressed in reporting medical education research. It thus provides a classification taxonomy and describes research settings and outcomes more precisely. It serves both as a potential synthesis framework as well as a blueprint guideline for reporting outcomes. This will support researchers in medical education and their and reviewers alike. We outline potential next steps to further validate and advance our model. It will facilitate the routine reporting of outcome levels.

**Supplementary Information:**

The online version contains supplementary material available at 10.1186/s12909-026-08579-z.

## Introduction

Although Chen et al. made a call for reporting outcome levels in medical education research more than 15 years ago and introduced a well-researched theoretical model, it has not yet been integrated into reporting standards [1]. However, there is a broad understanding that educational interventions need to produce meaningful outcomes, i.e., have a positive impact not only on learners’ satisfaction, but, ideally, on the overall quality of care. With the introduction of the MERSQI (Medical Education Research Study Quality Instrument), a tool intended to measure the quality of research in medical education, Reed et al. called for a shift towards the reporting of outcomes measures to enhance the quality of research and provide insights into the effectiveness of interventions [2]. Similarly, several other publications also discuss the importance of outcome reporting in medical education research and reporting as early as 1990 [3–6].

Frye and Hemmer’s discussion of appropriate evaluation models to choose from when evaluating medical education programs provides a clear framework to report such outcomes in scientific literature as they call for models with enough complexity to adequately depict medical education [7]. However, none allows for the reporting of all outcomes relevant in health professions education.

Kirkpatrick’s four-level model is a robust model widely used in the evaluation of educational programs and can therefore also guide outcome reporting in scientific papers or project reports [8, 9]. While it is easy to understand and use, it falls short on the representation of the complexity of medical care particularly when reporting the results of medical education ranging from patient care to health policy on an organizational level. The easiness of use comes with the price of limited granularity, as the model might lead to the oversimplification of outcomes into the four broad categories of reaction, learning, behavior, and results. This lack of detail makes it difficult to capture the nuanced, multi-dimensional aspects of medical education, particularly in settings where outcomes may not neatly fit into one of these levels. Particularly in terms of its level 4, the “why” aspect, i.e., the practical effect or effects at an organizational level. There is no clear differentiation between patient satisfaction, patient health, general quality of care, or even effects at a higher level. All of these points would be summarized under Kirkpatrick’s Level 4. Additionally, it is mostly designed for postgraduate training, and falls short in dealing with pre-qualification education.

The above mentioned model by Chen et al. [1] has a strong theoretical background and includes physician/learner outcomes as well as patient outcomes. However, it does not allow for the reporting of learner satisfaction. The complex depiction of the framework makes it challenging to use and to report outcomes. A model to specifically design and assess continuous medical education programs by Moore et al. names learners’ satisfaction, physicians’ competence and performance as well as patient-centered outcomes but omits organizational-level outcomes [10]. The latter is considered in a model by O’Malley et al., but this model fails to integrate learner satisfaction [11].

While learner satisfaction alone might not be a sufficient marker for the success of an educational activity, it corresponds to a higher likelihood of successful completion of a study program and a higher sense of preparedness for the profession [12, 13]. Learner satisfaction has been and still is one of the most common reported outcomes in medical education literature and thus has to be included in a comprehensive outcomes-reporting model [14, 15].

Furthermore, the above-mentioned models tend to prioritize quantitative outcome measures, with limited capacity for qualitative research. Medical education, however, often involves complex, context-dependent interventions where qualitative data can provide rich insights into learner experiences, contextual factors, and implementation challenges.

Neither model allows for descriptions of educational programs or interventions that do not yet have any outcome data. However, in many instances, descriptions of development or implementation processes are the data reported in medical education literature. A systematic review of the use of Entrustable Professional Activities – work-unit tasks or responsibilities in professional practice that a learner can be trusted to perform independently once they have demonstrated sufficient competence – clearly shows that, especially in the first years after its introduction the majority of articles focused on development and implementation [16–18]. Articles reporting outcome data as specified in the above-mentioned models only emerged in subsequent years.

Nevertheless, we believe that those reports are highly valuable for the medical education community. They provide clear guidance on potential pitfalls and effective strategies for adopting new approaches to guide others. Through the incorporation of implementation reports, processes can be better understood and replicated [19]. The understanding of how a program was implemented and which factors – such as training, feedback, or adaptation throughout – played a role can aid future implementation [20]. Moreover, those factors can be crucial for the sustainable implementation of a program [21].

Narrative reports provide data at low level expense to allow understanding specific reflections on developmental processes, and explaining for perceived associations to make them comprehensible. This may be helpful when developing new research questions, concepts or models in medical education.

These perspectives highlight the importance of including implementation-focused reports as part of the evidence base in medical education, offering valuable context that guides future efforts, even before definitive outcome data is available.

Another approach to review medical education literature is proposed by Harden et al. in the first Best Evidence in Medical Education (BEME) Guide [22]. Using the acronym QUEST, it is tailored for making assessments about the quality of evidence, its utility, extent, strengths and targets. While this approach allows for the inclusion of all types of evidence, including descriptive and qualitative studies, and can be a helpful tool, its primary aim is not to report outcomes but to assess the process of generating evidence.

Against this background, we propose the development and testing of a simple, easily useable and understandable model serving both as a reporting guideline as well as a framework for assessing outcomes of educational interventions levels in medical education. This model will build on existing models and address their weaknesses. We aim for a model that can be easily used both to promote the habitual reporting of outcome levels, as well as to assess them in systematic reviews.

## Methods

**Fig. 1:**
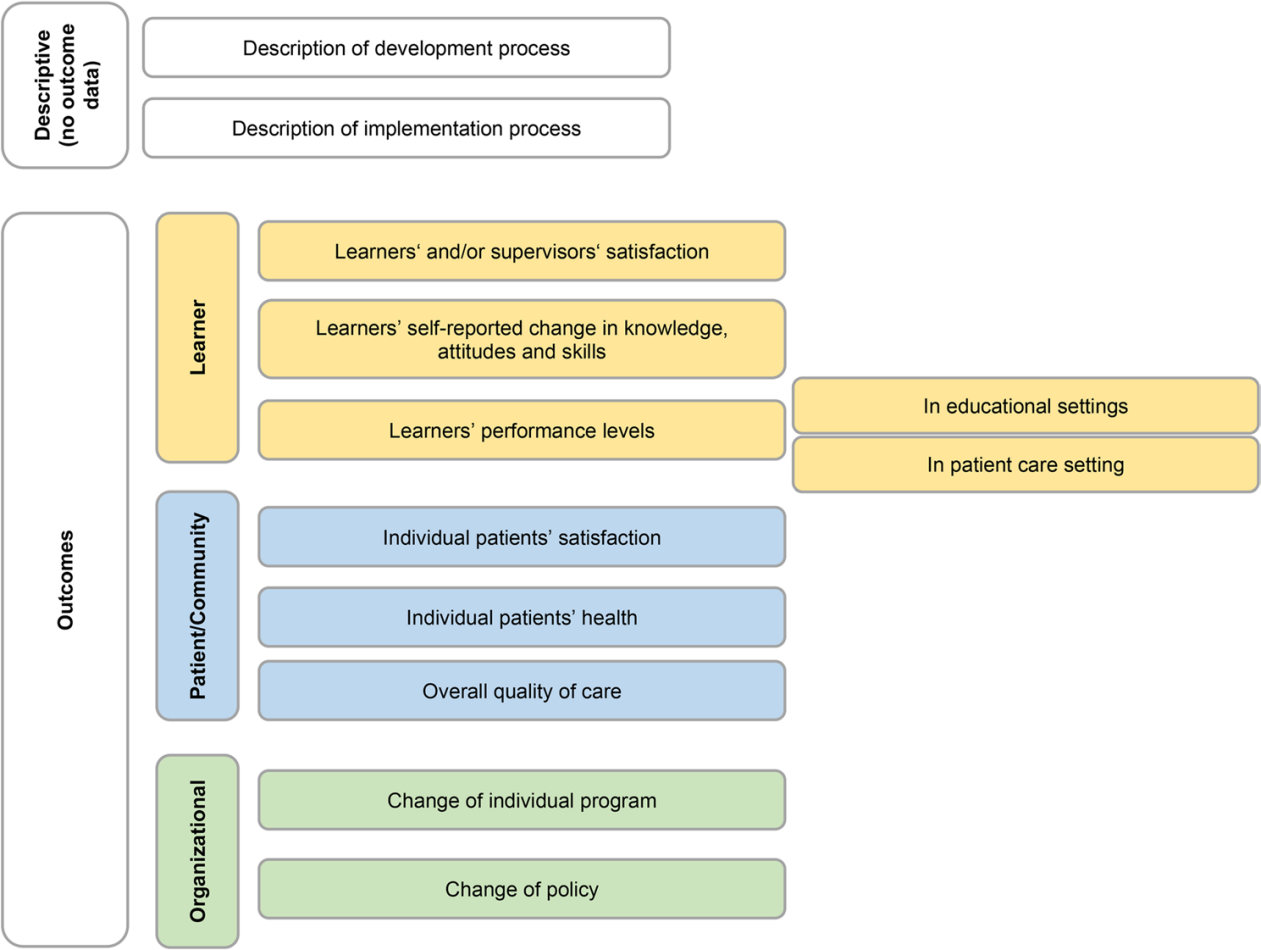
The final model depicting the outcomes reported in medical education literature

A modified double diamond approach was used for the development of the new model (Fig. 1). After the lack of a suitable, easy-to-use model for reporting outcomes relevant to medical education was identified, existing models for reporting outcomes in (medical) education were analyzed by two authors (JLK, HMB) regarding their strengths and weaknesses and further outcomes relevant to medical education were identified. An overview of the adopted concepts from each of the pre-existing models is provided in Table 1. Two authors (JLK, HMB) then drafted a model depicting the possible outcomes reported in medical education literature (Supplementary Material 1). We based this framework on the model proposed by Chen et al. [1], the Seven-Level Outcomes Model used by the American Academy of CME (Continuous Medical Education) [10], and the framework to measure the effectiveness of interventions in training health-care workers [11] as well as Kirkpatrick’s four-level model [8].

To test the applicability of the model, we aimed for a group of raters (*n* = 6, RL, JWW, EL, RLM, MN, JJYY) including individuals both from medical education as well as from other fields, and a representation of researchers from different countries (Australia, Canada, Columbia Germany, United States). Each reviewer had a unique skillset that added expertise to the process. Five raters had a background in medical education, either in research or as physician educators. We strove to include both younger educators and researchers (less than ten years of experience, *n* = 3) as well as more experienced ones (more than 20 years of experience, *n* = 2). The three raters without a background in medical education added expertise in developing and working with frameworks and experience in systematically reviewing, evaluating, and integrating research findings. This approach of selecting the raters with a broad range of relevant knowledge, skills and experience was chosen to ensure that the model would be understood regardless of formal training in medical education and across different contexts.

**Fig. 2:**
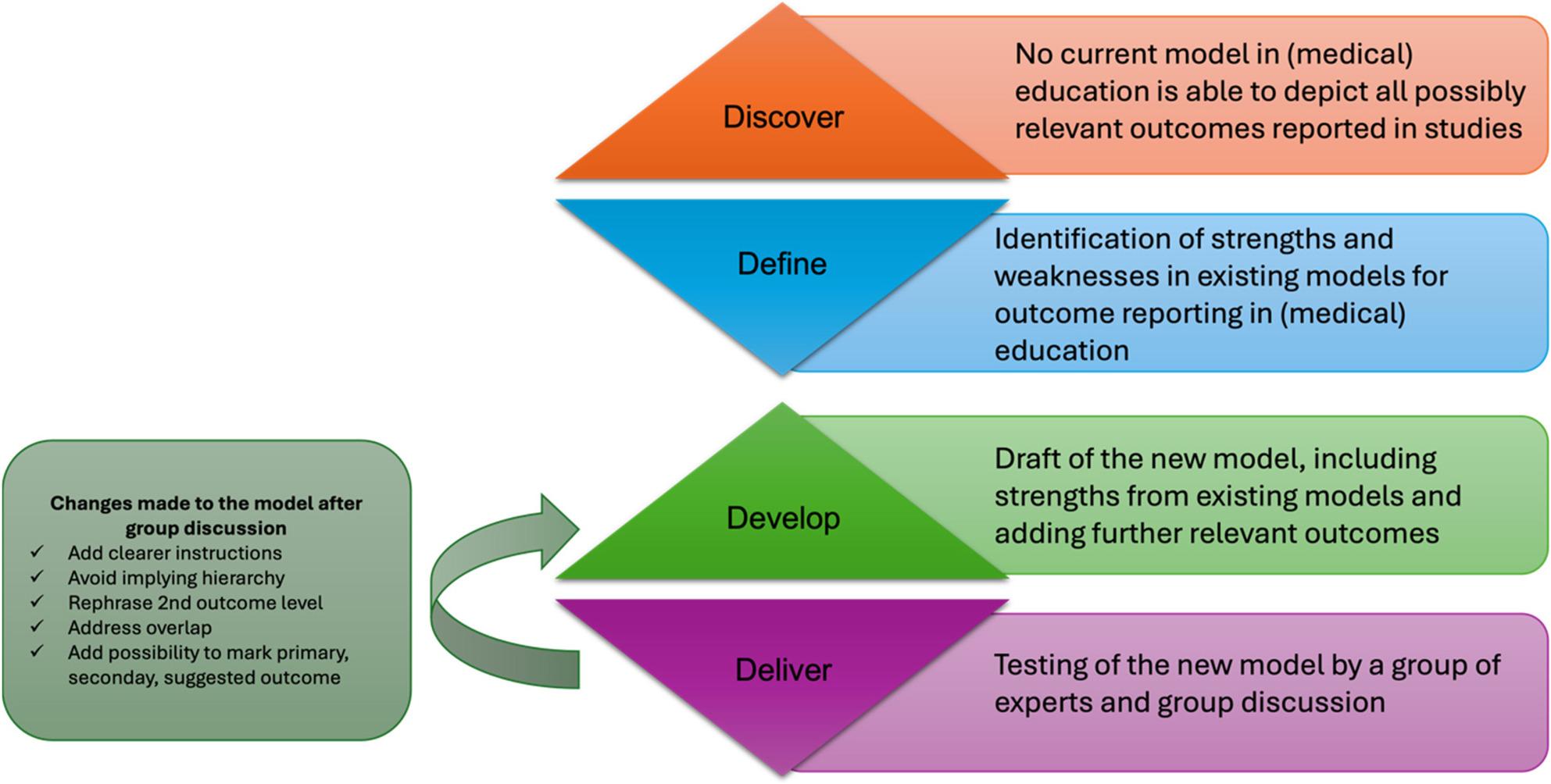
The development process of the ROME model

Although we decided not to calculate the inter-rater agreement at this stage as the initial rating was to be used to inform the revision of the model (Fig. 2), we followed the guidelines by Donner and Rotondi to determine sample size [23]. Assuming an excellent interobserver agreement, this led to the decision to have six raters evaluate 25 original research articles with our model.

To select a random sample of original studies from the field of medical education, we performed a search of Medline using the MeSH Terms: “Education, Medical” and limited the search to the years 2019–2021, English language, and full text available online. We chose the limitation for the years of publication to ensure that the model would be able to depict current developments in medical education. We excluded all non-original articles such as editorials, letters to the editor, or review articles. We imported all citations into an Excel sheet and used a random number generator to select 25 studies to be assessed.

We sent the draft model to the raters (*n* = 6, RL, JWW, EL, RLM, MN, JJYY) who used an Excel sheet to document the evaluation that incorporated the model. The Excel sheet was developed by one author (JLK) and reviewed by a second (HMB) prior to the rating process itself. Each rater independently assessed the randomly selected studies. We asked raters to keep notes on the rating process, the clarity of the instructions, appropriateness of categories, and the model in general. For each outcome, a rating of “yes” or “no” could be assigned indicating whether it was reported or not. The rating process including the Excel sheet, clarity of instructions, possible utilization in the reporting of one’s own as well as evaluation research of other raters, and usefulness were discussed between the raters in an online video meeting guided by key questions (see Table 2).

**Table 1:** Overview of the literature/models used for the development of our proposed model

Model	Short Description	Adopted Aspects
Chen et al. [1]	Theoretical base for researching and reporting outcomes in medical education; shows relationship between education, outcome measures on the physician and patient levels.	- outcomes on student/physician level (i.e., knowledge, skills, behavior, attitudes) - patient outcomes
Kirkpatrick [8, 9]	Widely known and used model to measure interventions in education; hierarchical outcomes (reaction, learning, behavior, results); non-specific to medical education.	- adapted to medical education - different aspects of learner outcomes (reaction = learner satisfaction; learning = changes in knowledge, skills and attitudes; behavior = performance levels) - patient and organizational outcomes are the specific results of medical education
Moore et al. [10]	Model for outcomes of continuous medical education including outcome levels on the individual and organizational level.	- inclusion of learner and patient centered outcomes as well as organizational outcomes
O’Malley et al. [11]	Complex model describing outcomes for in-service training to be adapted for specific health care/public health training aspects; includes individual, patient and population level.	- inclusion of all outcome levels (individual = learner, population = organizational)

The group discussion was held online and video recorded with four of the co-authors in August 2023. Disagreements between raters were to be reconciled by discussion led by the moderator. We asked raters unable to attend to provide a brief written statement about their experience using the tool, as well as its perceived utility and applicability. One of the co-authors (HMB) led the discussion, while another (JLK) reviewed the video. Both took field notes and discussed the results afterwards. The revised model was to be sent to all raters for agreement. Revisions were to be made until all raters agreed, if necessary, another group discussion was to be scheduled.

## Results

### Group discussion

All raters either attended the group discussion (*n* = 4) or provided feedback (*n* = 2). The group agreed unanimously that the tool was easy to understand and use, i.e., no formal training in medical education was needed and the tool does not require elaborate instructions. Raters felt that the Excel sheet was easy to use and well suited to guide the process. There were no further comments or requests to change the sheet. However, a short introduction or instructions on how to use the tool should be provided. Participants discussed whether the design of the model implied a hierarchical structure that was not necessarily justified.

Some studies reported several outcomes as primary and secondary endpoints and discussed possible effects on other outcomes which could not be adequately represented. It was unclear whether and if so, how, overlap was supposed to be indicated. For example, a study might measure learners’ performance levels in an examination (which would be the primary endpoint) but also ask learners about their experience (the secondary endpoint) and argue that this intervention might lead to better patient satisfaction. The group discussion and the written statements revealed that the categories were not always distinct or sharply discriminative, particularly when categorizing qualitative studies. Nonetheless, all raters agreed that aspects of qualitative studies could be well identified and categorized.

The group was unanimous in its agreement that the framework had high face validity and would generate reproducible results. While no formal inter-rater agreement was calculated in this study, the ratings submitted prior to the group discussion demonstrated close similarity among raters. In almost all cases (96%), the majority of raters agreed at least on the broader category for the reported outcomes (i.e., descriptive only, learner, patient/community, or organizational). When there was ambiguity, agreement tended to be slightly higher amongst those raters with a medical education background. Some disagreements, however, were due to the initial lack of clarity as to whether two or more categories could be rated “yes” for the same study. The category with most agreement was the patient/community outcome level. In all cases, the majority (i.e., at least 5 out of 6) raters agreed if a sub-category was not reported. The agreement for the majority of raters for the positive reporting of a category was 80%. Raters were not asked to resolve discrepancies through discussion, but the consensus in the group discussion was that clearer instructions would facilitate better agreement.

Raters noted the applicability of the model to studies reporting both quantitative and narrative outcomes as well as those that have no measured outcomes yet such as project reports. Raters agreed that qualitative studies could also be assessed using the model. There were no substantial disagreements between raters during the group discussion. No additional disagreements were raised in the written statements.

### Revision of the model

Complying with the outcomes of the group discussion, we made some changes to the initial model. However, as agreement was already high between raters, no significant changes were made, and the fundamental design of the model was kept. We reworked the color scheme depicting outcomes to avoid the implication of a hierarchy of outcomes and therefore replaced the initial use of “outcome levels” by simply “outcomes”. We rephrased the second learner outcome to clarify the difference between the two outcomes (i.e., self-reported changes in knowledge, skills, attitudes, and objectively measured performance). All co-authors reviewed and approved the revised model (Fig. 2) and Excel sheet (Suppl. 3). No additional round of discussions was necessary. The revised model was named ROME – reporting outcomes in medical education – model.

### Development of the final rating sheet and descriptive examples

The Excel sheet was reworked after the group discussion. We added a drop-down menu as well as a short introductory text explaining the use. Raters suggested to add the possibility to indicate the primary and secondary outcomes in the Excel sheet as well as outcomes that are discussed or proposed but not measured. Thus, we added specific indicators to the Excel sheet to better classify different objectives of assessed studies (“1” indicating the primary outcome(s), “2” the secondary, and “S” suggested outcomes, see Suppl. 2). Following the result of the group discussion that a more detailed description would facilitate the easiness of use of the tool and its Excel rating sheet, we developed two illustrative examples (i.e., fictional studies) with the help of ChatGPT (OpenAI. ChatGPT (GPT-5). Aug 2023 version. Available from: https://openai.com/chatgpt) (Suppl. 3).

## Discussion

### Implications

Our framework for reporting outcomes in medical education offers a simple yet effective way to present research findings, making it accessible to both experts and those with limited prior knowledge. By requiring minimal explanation, it serves as a classification taxonomy allowing users to quickly grasp its structure and apply it effectively. It is intended to capture a broad spectrum of outcomes, from basic learner satisfaction to more complex clinical, institutional, and policy-level results – outcomes that are ultimately most critical in evaluating medical education programs [24]. Unlike other existing models, this framework incorporates all potential outcomes, providing a highly versatile tool applicable across a wide range of educational contexts. To avoid an implication of a hierarchy of outcomes we avoid the term „*outcome levels*” by simply using “*outcomes*”.

Naturally, in a specific study there often may be an overlap of outcome levels, particularly when primary and secondary outcomes are considered. Our model may map all outcomes of a study and is able to differentiate between primary and secondary outcomes. We suggest the primary endpoint should be chosen to generally classify a study. To further illustrate this, we have developed two use cases (Suppl. 4).

Given its alignment with Kirkpatrick’s widely recognized model, it facilitates rapid understanding, further enhancing its usability without prematurely assigning outcomes to a potentially higher or lower level [8]. In contrast to other models discussed above, it is intended to incorporate all possible outcomes making it highly versatile and suitable for use in a wide range of contexts particularly for more complex outcomes [7, 10, 11]. The provision of an Excel sheet that can be used easily with standard Office software facilitates the low-threshold use. Instructions and a fictional example included with the Excel sheet further aid the application of the model especially by those that do not have a strong background in medical education. The consensus among all members of the study group is that the model is sufficiently straightforward to identify major components, while simultaneously allowing for sufficient detail to capture the essence of an intervention. We suggest our model as guideline to standardize reporting outcomes in medical education.

### Limitations

Despite the strengths of the framework, some limitations need to be acknowledged. Not all raters were able to attend the online group discussions in person, although written statements were provided in these instances. Ultimately, a consensus on structure, items and validity of our model was reached in a subsequent round, but the lack of in-person engagement might have impacted the depth of discussion in some cases. Another key limitation is the need for formal validation of the framework’s inter-rater reliability. Although our preliminary analysis showed little disagreement among raters, additional research is needed to formally assess the framework’s consistency and reliability across different raters and contexts.

### Future research

Moving forward, several avenues for future research could refine and validate our framework. We recommend that future studies build on the process we described, again selecting raters from different fields and different cultural backgrounds, including the calculation of an inter-rater reliability. Based on our calculations assuming an excellent interobserver agreement of Fleiss’ kappa = 0.8 – this assumption being supported by our preliminary findings –, a presumed prevalence rate (pi) of 0.3 and aiming for a lower bound of the 95% confidence interval of kappa = 0.6 (corresponding to at least a substantial interrater agreement [25]), this could be reinvestigated by having six raters assess 25 original research articles again. Considering the preliminary findings after comparing the tables filled by the raters which showed little disagreement, we are confident that 25 original articles were a sufficient number to test the framework’s general usability and face validity.

Another promising approach could be to apply established methods like the Delphi technique to gather more comprehensive feedback from a broader range of experts. This could help fine-tune both the framework’s usability and its theoretical foundation. Furthermore, more in-depth studies are needed to explore how evidence from medical education interventions is actually implemented in practice and policy.

### Outlook

Ellaway et al. point out that little is known – or reported in the literature – on how evidence on educational interventions are actually applied in practice and policy [26]. A study by Thomas et al. found that educators generally are favorable of applying evidence-informed educational interventions, but there seem to be barriers to the reporting and reception of such evidence [27]. In another article, they argue that in order to be impactful, research in medical education should be conducted in the context in which it will most likely be used and in the environment it will affect [28]. Additionally, using the framework could enhance communication among medical educators, especially those without formal training in educational theory.

A wider adoption of this framework could help standardize the reporting of outcomes in both individual studies and systematic reviews, ultimately improving the quality and impact of medical education research.

**Table 2:** Key questions for the online group discussion

1. Please describe your experience with the proposed framework for reporting outcomes.
2. Do you feel that the categories and instructions are clear?
3. Do you think the categories adequately represent outcomes reported?
4. Do you think rating the categories is feasible and generates reproducible results?
5. Is such a framework helpful for evaluating research of others?
6. Would such a framework be helpful to report outcomes of your own research?
7. Do you think the framework might be helpful to assess quality of a publication?
8. Please describe any changes that you would like to make to the framework.

## Supplementary Information

Supplementary Material 1.

Supplementary Material 2.

Supplementary Material 3.

## Data Availability

The video material is not publicly available due to privacy reasons. Other than that, data sharing is not applicable to this article as no datasets were generated or analyzed during the current study.

## Abbreviations

ROME model: Reporting Outcomes in Medical Education model
CME: Continuous Medical Education
