# Supplementary material for: The reporting outcomes in medical education (ROME) model: proposition of a new framework
Source: BMC Med Educ. 2026 Jan 12;26:152. doi: 10.1186/s12909-026-08579-z (PMC12849325; doi:10.1186/s12909-026-08579-z)

Supplementary Material 3: Fictional Illustrative Studies With Instructions For The Use of The Excel Rating Sheet

Fictional Study 1: Peer-Led Education Intervention to Enhance Clinical Skills in Undergraduate Medical Students: A Learner Satisfaction Study

Introduction:

Clinical skills are essential for medical students, but traditional teaching methods can limit peer interaction. This study explores the impact of a structured peer-led intervention aimed at improving clinical skills and measures student satisfaction with the process. We hypothesized that students would report high satisfaction and perceive the intervention as beneficial for skill development.

Methods:

Study Design:

This prospective study was conducted at a large medical school during the Spring semester of 2024. A total of 120 second-year undergraduate medical students participated in the intervention. The study was approved by the institution's ethics committee.

Intervention Development Process:

The development of the peer-led clinical skills intervention followed a structured process to ensure educational effectiveness and practical implementation.

1. Needs Assessment:

Surveys and focus groups with second-year students revealed challenges with current clinical training, such as limited hands-on practice and a lack of collaborative learning. The peer-led model was chosen to address these concerns by offering a supportive, low-stakes environment.

2. Curriculum Design:

A curriculum focusing on key clinical skills, including patient history-taking, physical exams, and communication, was designed. Faculty collaborated with senior students to ensure content was relevant and accessible. Each session combined brief lectures with hands-on practice using simulated patients.

3. Peer Educator Training:

Senior students were trained in teaching methods, giving constructive feedback, and managing group dynamics. The training was led by faculty and focused on creating a collaborative and effective learning environment.

4. Simulated Patient Preparation:

Simulated patients were trained to portray consistent clinical scenarios. Faculty and simulated patients worked together to refine scenarios and ensure realistic interactions, offering students a safe environment for practice.

5. Feedback Mechanisms:

A structured feedback rubric focused on clinical skills, communication, and critical thinking was developed. Peer educators were trained to provide specific, actionable feedback in a supportive manner, fostering student improvement.

6. Implementation Logistics:

Sessions were scheduled around students' timetables, with groups of 6-8 students per session. Faculty members oversaw each session, ensuring content accuracy and providing additional support as needed.

7. Evaluation and Adjustment:

Ongoing feedback from students and peer educators helped refine the intervention. Adjustments were made to session content and pacing to improve effectiveness and engagement.

The final intervention consisted of the following components:

1. *Peer-led Clinical Sessions:*
   Senior students (3rd and 4th year) were trained to facilitate peer-led clinical skills workshops, focusing on key procedures such as taking medical histories, performing physical exams, and delivering patient education. Each session was structured to include a brief lecture, followed by a hands-on practice session using simulated patients.
2. *Simulation and Practice:*
   Students participated in small-group practice sessions, with each group consisting of 6-8 students. Simulated patients were used to create realistic clinical scenarios where students could practice their skills. Each student had the opportunity to engage in direct patient interaction while peers observed and provided constructive feedback.
3. *Peer Feedback:*
   After each session, peers provided feedback based on structured rubrics that evaluated communication skills, clinical reasoning, and technical proficiency. Feedback was delivered in a supportive environment with the aim of fostering improvement and peer learning.
4. *Faculty Oversight:*
   Faculty members were present during each session to provide oversight and ensure the accuracy of the clinical content. They also provided additional guidance and addressed any clinical questions raised by students.

Data Collection:

Learners' satisfaction with the intervention was assessed using a structured questionnaire administered at the end of the intervention period. The questionnaire included both Likert-scale items (ranging from 1 = strongly disagree to 5 = strongly agree) and open-ended questions. The survey covered several domains:

- Perceived usefulness of the peer-led sessions in developing clinical skills
- Effectiveness of peer feedback in improving individual performance
- Satisfaction with the learning environment and overall experience
- Level of confidence in performing clinical skills after the intervention

Statistical Analysis:

Quantitative data from the Likert-scale items were analyzed using descriptive statistics to determine overall satisfaction levels. Comparisons between different student groups (e.g., based on prior experience) were performed using t-tests and ANOVA, as appropriate. Qualitative data from open-ended responses were analyzed thematically to identify common themes.

Results:

Out of 110 respondents (92% response rate), the following findings emerged:

1. *Satisfaction with Peer-Led Sessions:*
   - 85% felt the sessions improved their clinical skills.
   - 88% appreciated the collaborative learning environment.
2. *Effectiveness of Peer Feedback:*
   - 80% found peer feedback helpful for improvement.
   - 75% felt more confident performing clinical tasks post-intervention.
3. *Overall Experience:*
   - 92% were satisfied with the overall experience, with many noting the supportive and hands-on nature of the learning environment.
4. *Confidence in Clinical Skills:*
   - 87% felt more confident in history-taking and physical examination.

Qualitative Themes:

Students valued the opportunity to learn in a low-pressure environment with immediate feedback. Peer-led sessions were seen as less intimidating than instructor-led sessions.

Discussion:

The study suggests that peer-led education is effective in enhancing clinical skills and fostering a positive learning environment. Students reported high satisfaction with the intervention, highlighting the value of peer feedback and collaborative learning. The results support the integration of peer-led sessions into medical curricula, offering an alternative to traditional methods.

Conclusion:

Peer-led education can enhance clinical skills and learner satisfaction in undergraduate medical education. The model promotes confidence, collaboration, and active learning, making it a valuable addition to medical training programs.

Comment: This study describes the development and implementation process of a peer-led educational intervention for medical students. The success of the program is measured by learners' satisfaction and self-reported confidence in clinical skills (i.e., self reported change in skills). It includes both quantitative and qualitative results. The categories are highlighted in the model below.

Using the Excel sheet to report or assess outcomes for this study, you would select "yes" for those outcomes and "no" for all others.

Alternatively, if you choose to indicate primary and secondary outcomes, you might choose learners' satisfaction and self reported change in skills as primary ("1") and the descriptive outcomes as secondary ("2"). Furthermore, the discussion hints at the possibility of a permanent change in the curriculum without directly reporting this. Thus, you might indicate the "change of individual program" or "change of policy" with "S" for "suggested outcome". This should reflect the intention of the study's authors, not a judgment made by you! The numbers assigned do not imply a hierarchy nor add up to a score of any kind.

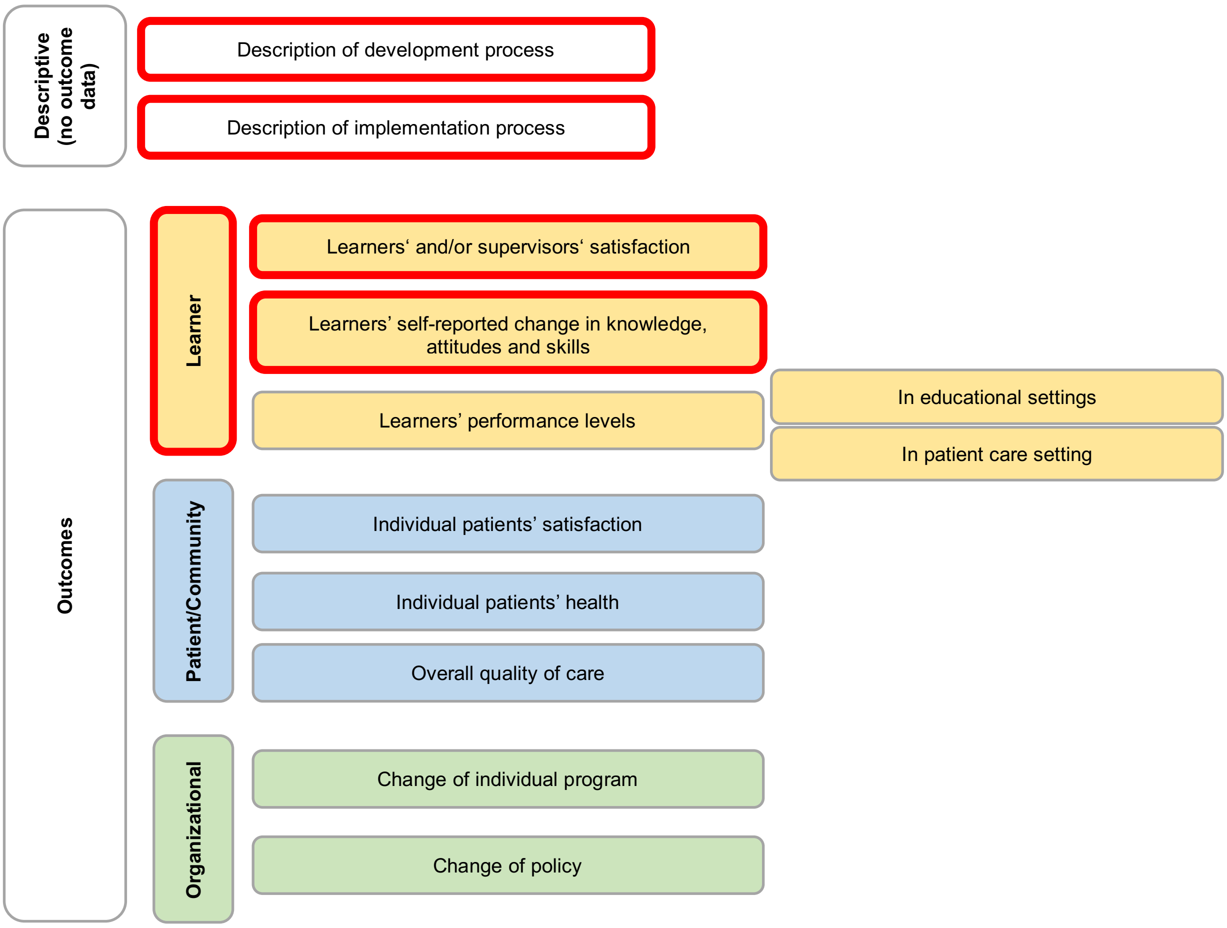

Fictional Study 2: Assessing the Impact of a Patient-Centered Education Intervention on Medical Students' Clinical Decision-Making and Patient Health Outcomes: A Curriculum Change at XYZ Medical School

Introduction:

Medical education traditionally emphasizes technical knowledge and clinical skills, but recent research suggests that patient health outcomes are also influenced by factors such as communication, empathy, and shared decision-making. At XYZ Medical School, there was a growing recognition that medical students could benefit from a stronger focus on patient-centered care. This study aimed to assess whether a targeted educational intervention that focused on these aspects could improve both students' clinical decision-making and patient health outcomes, ultimately influencing curriculum changes.

Methods:

Study Design:
This prospective, controlled intervention study was conducted at XYZ Medical School during the 2024 academic year. The intervention was delivered to 100 third-year medical students, and patient health outcomes were tracked over a six-month period following the intervention.

Intervention:
The intervention focused on improving students' patient communication, decision-making skills, and empathy through the following components:

1. Workshops on Patient-Centered Care: Students attended weekly workshops that included interactive case studies and role-playing exercises aimed at improving communication and shared decision-making.
2. Patient Interaction Simulations: Using standardized patients, students practiced discussing treatment options, involving patients in decision-making, and addressing patient concerns.
3. Mentorship and Reflection: Students were paired with experienced clinicians who guided them through reflective practice, encouraging empathy and focusing on the patient's perspective in clinical scenarios.

Patient Health Outcome Measurement:
Patient health outcomes were measured through:

- Clinical Indicators: Pre- and post-intervention assessments of patients' health, including symptom reduction, medication adherence, and recovery progress. Data were collected from patients in clinical rotations where students had direct involvement in care.
- Patient Satisfaction: Patient-reported satisfaction with communication, involvement in care decisions, and overall experience with the healthcare team.
- Follow-up Data: Health outcomes were measured over a 3-month follow-up period, including symptom monitoring and healthcare utilization (e.g., hospital readmission rates).

Control Group:
A control group of 100 students who received traditional clinical training without the patient-centered education intervention was used for comparison.

Data Analysis:
Quantitative data (clinical indicators and patient satisfaction scores) were analyzed using paired t-tests and linear regression to compare outcomes between the intervention and control groups. Qualitative data from patient feedback were analyzed thematically to identify common themes related to the patient experience.

Results:

Out of 200 students, 190 completed the study (95% response rate), with 100 students in the intervention group and 90 in the control group. The following results were observed:

1. *Clinical Decision-Making:*
   - Students in the intervention group demonstrated improved clinical decision-making. 85% of intervention group students made decisions that aligned more closely with evidence-based guidelines and patient preferences compared to 70% in the control group ($p < 0.05$).
2. *Patient Health Outcomes:*
   - Symptom Management: 72% of patients whose care involved intervention students reported significant improvement in their symptoms, compared to 55% in the control group ($p < 0.05$).
   - Medication Adherence: Patients cared for by intervention students showed a 20% higher rate of medication adherence (85% vs. 70%, $p = 0.03$).
   - Follow-up Outcomes: There was a 15% reduction in hospital readmissions among patients treated by intervention group students compared to those treated by control group students ($p = 0.04$).
3. *Patient Satisfaction:*
   - Patient satisfaction scores were higher for those treated by students in the intervention group. 92% of patients reported feeling involved in their care decisions, compared to 70% in the control group ($p = 0.02$).
   - 88% of patients felt their concerns were adequately addressed by students in the intervention group, compared to 66% in the control group ($p = 0.03$).
4. *Qualitative Feedback:*
   - Common themes from patient feedback included appreciation for clear communication, feeling more confident in their care decisions, and a greater sense of empathy from students.
   - Students in the intervention group reported feeling more engaged in patient care and valued the opportunity to practice patient-centered communication skills.

Discussion:

The results of this study suggest that the patient-centered education intervention significantly improved clinical decision-making and patient health outcomes. Students in the intervention group made more accurate, patient-centered decisions and achieved better outcomes in terms of symptom management, medication adherence, and follow-up health. The positive impact on

patient satisfaction further emphasized the importance of effective communication and shared decision-making in clinical practice.

Given these results, XYZ Medical School made the decision to revise its curriculum, integrating patient-centered care principles into all stages of medical education. This curriculum change emphasizes:

- Enhanced training on communication skills across clinical years
- Regular use of standardized patients for practicing shared decision-making
- Continuous reflection on empathy and patient experiences in clinical settings

The intervention demonstrated that patient-centered education not only benefits medical students' clinical skills but also leads to tangible improvements in patient health outcomes, reinforcing the need for such educational approaches in medical curricula.

Conclusion:

The patient-centered education intervention significantly improved clinical decision-making and patient health outcomes. As a result, XYZ Medical School has integrated these principles into its curriculum, reinforcing the importance of patient communication, empathy, and shared decision-making in medical training. This shift aims to produce more holistic, compassionate healthcare professionals who are better equipped to meet patients' needs.

Comment: This study describes an intervention in a medical school. While there is a short paragraph on the project itself, neither the development nor the implementation process is described in detail. To assess the success of the intervention, outcomes on the patient/community level are reported: individual patients' satisfaction and health. As a result, the change of the curriculum (i.e., individual program) at the medical school is reported.

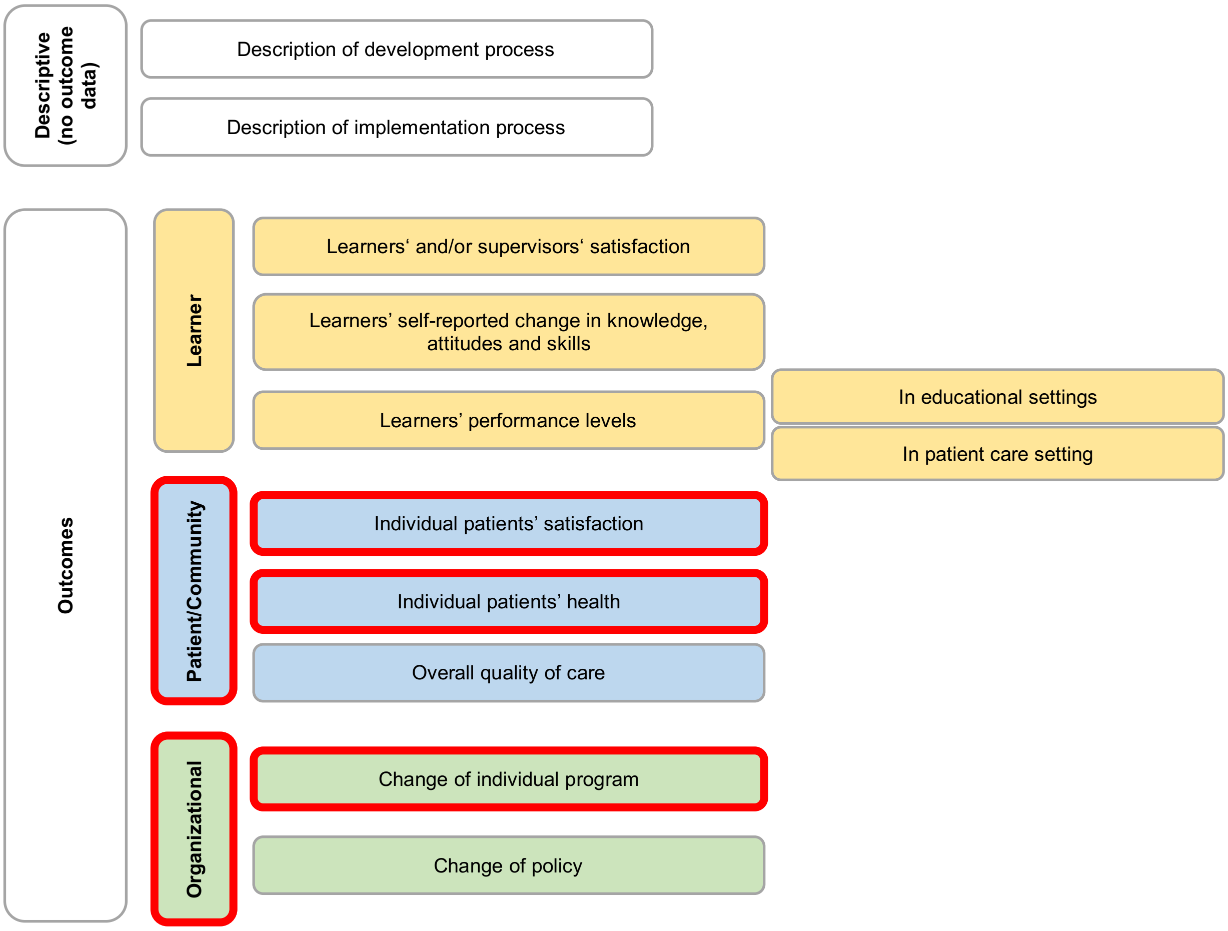

Supplement: Supplementary file 2 — Supplementary Material 2. [file 12909_2026_8579_MOESM2_ESM.pdf]
