# Supplementary figures and images for: The reporting outcomes in medical education (ROME) model: proposition of a new framework
Source: BMC Med Educ. 2026 Jan 12;26:152. doi: 10.1186/s12909-026-08579-z (PMC12849325; doi:10.1186/s12909-026-08579-z)

Supplementary Material 1. Initial model as proposed to the raters before group discussion.

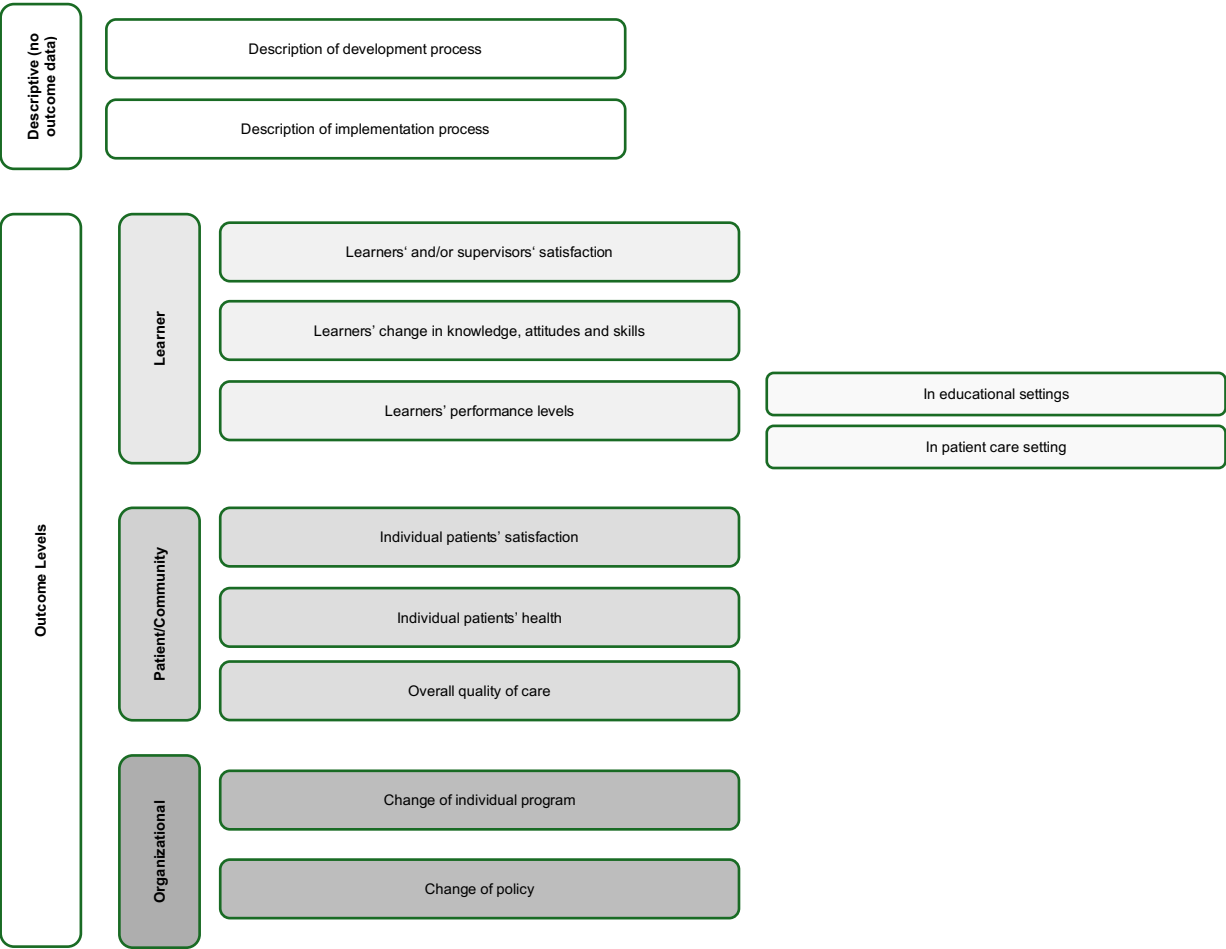

Supplement: Supplementary file 3 — Supplementary Material 3. [file 12909_2026_8579_MOESM3_ESM.pdf]
